# Targeting metastatic breast cancer with peptide epitopes derived from autocatalytic loop of Prss14/ST14 membrane serine protease and with monoclonal antibodies

**DOI:** 10.1186/s13046-019-1373-y

**Published:** 2019-08-19

**Authors:** Ki Yeon Kim, Minsang Yoon, Youngkyung Cho, Kwang-Hoon Lee, Sora Park, Se-ra Lee, So-Young Choi, Deokjae Lee, Chansik Yang, Eun Hye Cho, Sangjun Davie Jeon, Seok-Hyung Kim, Chungho Kim, Moon Gyo Kim

**Affiliations:** 1 Department of Biological Sciences, Inha University, Inharo 100, Michuhol-Gu, Incheon, Republic of Korea; 2 Division of Life Sciences, Seoul National University, Seoul, South Korea; 3 New Drug Development Center, Osong Medical Innovation Foundation, Cheongju, South Korea; 4 MedyTox, 114, Central town-ro, Yeongtong-gu, Suwon, South Korea; 5 Department of Pathology, College of Medicine, Sungkyunkwan University, Samsung Medical Center, Seoul, South Korea; 6 Department of Life Sciences, Korea University, Seoul, South Korea; 7 Convergent Research Institute for Metabolism and Immunoregulation, Inha University, Incheon, South Korea

**Keywords:** Prss14, Metastasis, Immunotherapy, Cancer vaccine, Autocatalytic loop

## Abstract

**Background:**

In order to develop a new immunotherapeutic agent targeting metastatic breast cancers, we chose to utilize autocatalytic feature of the membrane serine protease Prss14/ST14, a specific prognosis marker for ER negative breast cancer as a target molecule.

**Methods:**

The study was conducted using three mouse breast cancer models, 4 T1 and E0771 mouse breast cancer cells into their syngeneic hosts, and an MMTV-PyMT transgenic mouse strain was used. Prss14/ST14 knockdown cells were used to test function in tumor growth and metastasis, peptides derived from the autocatalytic loop for activation were tested as preventive metastasis vaccine, and monoclonal and humanized antibodies to the same epitope were tested as new therapeutic candidates. ELISA, immunoprecipitation, Immunofluorescent staining, and flow cytometry were used to examine antigen binding. The functions of antibodies were tested in vitro for cell migration and in vivo for tumor growth and metastasis.

**Results:**

Prss14/ST14 is critically involved in the metastasis of breast cancer and poor survival rather than primary tumor growth in two mouse models. The epitopes derived from the specific autocatalytic loop region of Prss14/ST14, based on structural modeling acted as efficient preventive metastasis vaccines in mice. A new specific monoclonal antibody mAb3F3 generated against the engineered loop structure could reduce cell migration, eliminate metastasis in PyMT mice, and can detect the Prss14/ST14 protein expressed in various human cancer cells. Humanized antibody huAb3F3 maintained the specificity and reduced the migration of human breast cancer cells in vitro.

**Conclusion:**

Our study demonstrates that Prss14/ST14 is an important target for modulating metastasis. Our newly developed hybridoma mAbs and humanized antibody can be further developed as new promising candidates for the use in diagnosis and in immunotherapy of human metastatic breast cancer.

**Electronic supplementary material:**

The online version of this article (10.1186/s13046-019-1373-y) contains supplementary material, which is available to authorized users.

## Background

Prss14 (serine protease 14)/ST14 (suppression of tumorigenicity 14), also known as epithin [1], matriptase [2], and membrane-type serine protease 1 (MT-SP1) [3], is the representative member of type II transmembrane serine proteases [4–6].

Multiple pathologic analyses of cancer patients’ samples showed that over-expression of Prss14/ST14 is consistently found in progressed cancers of epithelial types (reviewed in [5, 7]). Our careful examination on post surgery esophageal squamous cell carcinoma patients showed that the prognosis of disease free survival is extremely poor with Prss14/ST14 expression [8]. Its high level of expression was detected in various human breast cancer cell lines [9, 10] and breast cancer tissues [11–14]. The importance of Prss14/ST14 in breast cancer progression, metastasis, and patient’s survival is now clear. Recently, we reported that Prss14/ST14 is an excellent prognostic marker for estrogen receptor negative (ER^−^) or triple negative (TN) breast cancer type after systemically analyzing the signature genes of breast cancer types (ER, PR, HER2) and the epidermal-mesenchymal transition (EMT) genes using public gene expression data bases [8].

There are multiple genetically modified mouse models that exhibit phenotypes of Prss14/ST14 associated cancer. When the K5 promoter is used in transgenic expression of Prss14/ST14, mice spontaneously develop skin adenoma that can be accelerated by tumor promoting chemicals [15]. This study showed clear oncogenic function of Prss14/ST14. When MMTV-PyMT mouse models crossed with the matriptase hypomorphic mouse model [16], animals showed less breast cancer tumor burden and lived longer than the ones with normal levels of Prss14/ST14 expression [17]. This study also showed that Prss14/ST14 plays a critical role in cMet signaling in response to HGF secreted from the fibroblast in the tumor microenvironment.

The cellular functions of Prss14/ST14 for cancer progression and metastasis have been studied extensively using Prss14/ST14 high expressing cells and mouse models. 1) Ectodomain shed from the cell into culture media induced angiogenesis, such as endothelial cell migration, invasion and tube formation [18]. This angiogenic process can be blocked by specific polyclonal antibodies. 2) Prss14/ST14 is necessary and sufficient for EMT [19]. TGFβ induced EMT in the thymoma cell line 427.1.86 was blocked when Prss14/ST14 message was knocked down. In addition, when Prss14/ST14 gene expression was introduced, it led to EMT in MDCK cells. 3) Prss14/ST14 is critically involved in transendothelial cell migration of cancer cells via the upregulation of Tie2 signaling in the endothelium [20], and of macrophages upon IFNγ activation [21]. 4) Prss14/ST14 is important for 4 T1 breast cancer cells to form lung metastasis nodules in the intravenous tail vein injection model [20].

Many known specific substrates for Prss14/ST14 protease are well known for their roles in tumor progression and metastasis and can be categorized into multiple families [5, 7]. Extracellular matrix proteins including collagen and fibronectin can be degraded by the Prss14/ST14 protease activity. Therefore, the basement membrane is modified for easy cell infiltration during metastasis. Proteins involved in the tumor growth and proliferation such as EGFR [22] and PDGF-D [23, 24] are also the known substrates. Another family of proteins, such as MSP-1 [25] and Laminin 322 [26], are involved in cellular survival. In addition, Prss14/ST14 protease can activate multiple protease cascades by cleaving PAR-2 [27–29], uPA [9, 30], and MMP3 [31]. Prss14/ST14 protease activity can induce signaling by cleaving and activating either the cellular receptors or the ligands [7]. The capacity of auto activation of this protease [32, 33] is a key feature as an important part of the regulatory target to block tumor progression and metastasis.

In this report, we show that Prss14/ST14 is critically involved in lung metastasis of mouse breast cancer, and that an epitope containing the autocatalytic loop portion of the Prss14/ST14 protein can function as a preventive metastasis vaccine. A new monoclonal antibody specific for the autocatalytic loop recognizes the epitope in sequence and structure specific fashion and can reduce breast cancer cell migration and abrogate metastasis in MMTV-PyMT mouse model.

## Methods

### Cell and mouse

MCF7, T47D, MDA-MB-453, SNU216, MKN45, PC3, OE19, and HCT116 human cell lines were obtained from Dongeun Park (Seoul National University) and Jung Hwa Kim (Inha University). All cells were maintained in Dulbecco’s Modified Eagle’s Media (DMEM; Welgene) supplemented with 10% fetal bovine serum (FBS; Welgene or Gibco), penicillin and streptomycin (Welgene), and 4 mM L-glutamine (Welgene). All adherent cells were subcultured every 2~3 days with trypsin-EDTA (Welgene). Balb/c and C57BL/6 mice were purchased from Daehan Biolink. Mice were maintained in the Laboratory of Molecular and Cellular Immunology Animal Facility at Inha University and Korea Bio in Korea University in compliance with the use of Laboratory Animals under proper protocols. For orthotopic mouse breast cancer models, female Balb/c mice for 4 T1 cells or C57BL/6 for E0771 cells were anesthetized with Avertin (2,2,2-Tribromoethanol (Sigma) in 2-Methylbutanol-2 (Sigma)). The incision was closed with wound clips and the primary tumor outgrowth was monitored twice a week by taking measurements of the tumor length (a) and width (b). Tumor volume (V) was calculated by the formula determined by Carlsson: V = (ab^2^)/2, where ‘a’ and ‘b’ are the longest and shortest diameters of the tumor respectively [34]. For the antibody effect on the tumor model, female MMTV-PyMT mice at the age of 9 weeks old were intraperitoneally injected with PBS, Taxol, or mAb3F3 antibody with amounts at 50 μg/mouse. Treatment of five mice per group was initiated at 9 weeks old and injections were performed twice a week until 14 weeks of age. Tumor size was measured twice a week. Mice were euthanized at 15 weeks of age. The number and areas of nodules in the lung were measured by using ImageJ following photography.

### Antigenic peptide synthesis

Peptides were synthesized by Thermo or Abclone. The formation of a disulfide bond between the both ends was assessed via mass spectrometry (Abclone). Conjugation to keyhole limpet hemocyanin (KLH) or bovine serum albumin (BSA) was performed by Young In Frontier. Epi-Sc (EQGKGARDWPEWAVQGVNT), which has the same amino acid composition as the Epi-SP peptide (KQARVVGGTNADEGEWPWQ), was chosen using the web site (http://users.umassmed.edu/ian.york/Scramble.shtml) and synthesized by Young In Frontier.

### Protein structure modeling

The tertiary model structures of proteins and peptides were obtained from I-TASSER server (http://zhanglab.ccmb.med.unich.edu/I-TASSER/). The predicted models were analyzed by Chimera software (htt://www.cgl.ucsf.edu/chimera/download.html). Structure modeling of fragment antigen-binding (Fab) of the monoclonal antibody was performed by using PIGS web server (http://www.biocomputing.it/pigs). Antigen-Antibody docking modeling was obtained by ClusPro 2.0 web server (htt://cluspro.bu.edu).

### In vitro cell migration assays

For the wound healing migration assay, cells were grown to confluence. A linear mechanical scratch wound was generated using a blue tip. Cells were then incubated in low serum media. The wounded areas were observed under a microscope (IX51, Olympus). The samples were analyzed by wound healing tool in ImageJ software. For testing the antiserum in transendothelial migration assay, MS1 cells (5 X 10^4^) were grown on Boyden Chamber (8 μm pore insert, Falcon) until they reached to confluency. The 4 T1 cells were stained with CFSE (Molecular Probes) and resuspended with DMEM containing 5% FBS and anti-Epi-SP sera. The CFSE-stained 1 X 10^5^ 4 T1 cells were added onto the MS1 cells and incubated for 4 h. Cells were fixed with 3.7% formaldehyde, and remaining cells were scrubbed and washed. Images of CFSE-positive cells that migrated to the opposite side were obtained by using a microscope (IX51, Olympus) with AxioCam MRm (Zeiss). For testing purified antibodies, transwell migration assay was performed using Boyden Chamber. MCF7 cells (5 × 10^4^) were seeded on the upper chamber and incubated with antibody in serum-free media for 4 h. Then, the lower chamber was filled with 10% serum-containing media. After 24 h of incubation, the cells on the upper surface of the membrane were removed using cotton swabs. The cells on the lower surface of the membrane were fixed with 100% methanol for 10 min and stained with 0.2% crystal violet for 5 min. The migrated cells in ten fields from triplicate experiments were counted.

### Generation and humanization monoclonal antibody

Female Balb/c mice were immunized with a circular peptide conjugated with KLH, in Imject^R^ Alum (Thermo) four times at 3 week intervals. Three days before fusion, primed mice were boosted with the final immunization. At the day of fusion, a single-cell suspension from the harvested spleen was fused with SP2/0-Ag14 myeloma with 50% Polyethylene glycol (PEG) solution (Sigma) and plated in 96well plates with DMEM containing 20% serum. Hybridoma cells were selected in Hypoxanthine, Aminopterin, and Thymidine (HAT supplement) (Gibco) and maintained in Hypoxanthine and Thymidine (HT supplement) (Gibco). Hybridoma clones were selected by limiting dilution twice. For humanization, hybidoma originated antibody was humanized using a CDR-grafting method. The VH and VL sequences were searched for against the human germline sequence databases with IgBLAST (http://www.ncbi.nlm.nih.gov/igblast/) and IMGT/V-QUEST (http://www.imgt.org/IMGT_vquest/share/textes/), and the most similar human germ line Fv sequence and J region were identified. The residues within combined KABAT/IMGT CDR regions were grafted onto the framework regions of templates.

### Surface Plasmon resonance (SPR)

Binding of antibodies to the BSA conjugated peptide antigens were assayed using a Biacore T200 instrument (GE healthcare). Antibodies were injected for 60 s using a flow rate of 10 μl/min in the active flow cell only. For kinetic studies, antigens were diluted to the ranges of 1.25 to 20 nM. Signal detection was at a rate of 10 signals per second. Binding constants were determined using BIA Evaluation software version 1.0 (GE Healthcare).

### Enzyme-linked immunosorbent assay (ELISA)

For conventional ELISA, 50 ng of peptides in coating buffer (32 mM Na_2_CO_3_, 68 mM NaHCO_3_, 0.1% NaN_3_, pH 9.6) were coated on 96 well immunoplates (SPL), and blocked with 1% nonfat dry milk in PBS. After washing with 0.4 M Tris-Buffered Saline (pH 7.4) containing 0.1% Tween20, primary antibodies were added and incubated for 1 h at 37 °C. After washing, anti-mouse horseradish peroxidase (HRP) conjugated antibody was added and incubated for 1 h at 37 °C. Super AquaBlue ELISA substrate (eBioscience) was used for measuring optical density at 405 nm using ELISA Reader (TECAN, Sunrise™). Specific C-terminal coating ELISA was performed with peptide coating kit from TaKaRa by following the protocol provided. In the 96-well reaction plate, 225 ng of peptides in reaction buffer were coated with coupling reagent for 2 h at 37 °C. The coated wells were blocked for 1 h. Rest of the process was the same as for conventional ELISA except using distilled water as the wash buffer.

### Flow cytometry

To test mAb binding to native protein, HEK293T cells transfected with constructs (full length human Prss14/ST14, S805A mutant, and EGFP-S805A) were washed with PBS twice and treated with Enzyme-Free PBS-based cell dissociation buffer (Gibco) for single cell suspension. When CHO-S cells and HEK293TF cells were used, cells were harvested without dissociation. For analysis of mouse mAb3F3, 5 × 10^5^ CHO-S cells were used in FACS buffer (0.1 mg/ml BSA in PBS). To detect live cells, Fixable Viability dye eFluor 455UV (affymetrix) was treated for 30 min at 4 °C in dark. For cytoplasmic staining, cells were fixed and permeabilized with Fix & Perm kit (CALTAG) after washing. Human Fc receptor binding inhibitor (affymetrix) was used to block Fc receptors. mAbs were used as the primary antibody, PE conjugated anti-mouse Kappa (PharMingen) was used as a secondary antibody. Samples were filtered through 200 μm nylon mesh and read using FACSCalibur (BD) and analyzed using FlowJo software. For the analysis of humanized antibodies, HEK293TF cells were incubated for 3 days after co-transfection of pcDNA3.1/EGFP and pcDNA3.1/matriptase(S805A)-TST or of pcDNA3.1 and pCMV-tdTomato. The cells were washed with FreeStyle™ 293 Expression Medium. After blocking Fc receptors with blocking buffer (10% normal hamster serum, 10% normal rat serum, anti-FcR mAb2.4G2) for 5 min, the cells were fixed and permeabilized using Foxp3/Transcription Factor Staining Buffer Set (ThermoFisher) and stained with 30 μg/ml of humanized mAb3F3 antibody clones for 20 min. Alexa Fluor® 647 anti-human IgG Fc antibody (BioLegend) were incubated for 15 min for the secondary reagent. The samples were washed with media before reading using BD Accuri™ Flow Cytometer, and analyzed using Flowjo 10.

### Immunoprecipitation and western blotting

The cell lysates in IP buffer (150 mM NaCl, 1% Nonidet P-40, 1 mM EDTA, 50 mM HEPES, pH 7.4 and protease inhibitor cocktail) reacted with monoclonal antibodies overnight at 4 °C on a rotator and reacted to 50% slurry of protein A/G agarose bead (Pierce) for 2 h at room temperature on a rotator. The protein A/G beads capturing antibody-antigen complex were washed 3 times with PBS then mixed with SDS sample buffer and incubated for 5 min at 99 °C. The eluted supernatant was analyzed by western blot using PVDF membrane (Pall, FluoroTrans). After the membranes were incubated with 5% skim milk in 0.1% TritonX-100 in PBS (PBS-T), and reacted to polyclonal rabbit antibody IM1014 (Calbiochem) for human protein or polyclonal rabbit anti-epithin serum [35] for mouse protein. HRP-conjugated anti-rabbit IgG (1:5000 diluted) was used with Luminol/Enhancer solution and Stable Peroxide solution (SuperSignal^R^ West Pico, Thermo). The chemiluminescence was observed using LAS-4000mini (GE healthcare Life Sciences) and signals of each band were digitized by ImageJ.

### Immunofluorescence

MCF7, T47D, MDA-MB-453, SNU216, MKN45, PC3, OE19, HCT116, 427.1.86, and 4 T1 cells were seeded onto chamber slide or 0.1% gelatin coated coverslip, and fixed with 4% paraformaldehyde at room temperature for 10 min. Then the cells were permeabilized with 0.5% PBS-T and nonspecific binding was blocked with 10% goat serum/1% gelatin in 0.1% PBS-T for 30 min. mAb3F3 were used as primary antibodies. FITC-conjugated anti-mouse IgG was used as secondary antibody. Actin was visualized using phalloidin-iFluor647 (Abcam) and nucleus was stained with 0.3 μM DAPI (Molecular Probes) diluted in Mowiol mounting media (Sigma). These stained cells were visualized using confocal microscope LSM510 meta (Zeiss) and processed in Photoshop.

### Cell proliferation assay

For measuring proliferation of cells, 5 × 10^4^ of MCF7 cells were seeded in 12 well plates. The number of cells was counted at every 24 h using LUNA-FL™ Fluorescence Cell Counter (Logos). Acridine Orange and Propidium Iodide (Logos) were used to separate viable and dead cells.

### Cell cycle analysis

MCF7 cells were resuspended in 1.2 ml PBS and added 3 ml ice cold 95% ethanol dropwise while vortexing to fix the cells. The 1 × 10^6^ fixed cells were treated with RNase (100 μl/ml) and 400 μl Propidium Iodide (50 μg/ml) for 30 min at room temperature. The samples were read by FACSCalibur and analyzed by FlowJo.

## Results

### Prss14 is critical in metastasis of 4 T1 and E0771 mouse breast cancer cells

**Fig. 1:**
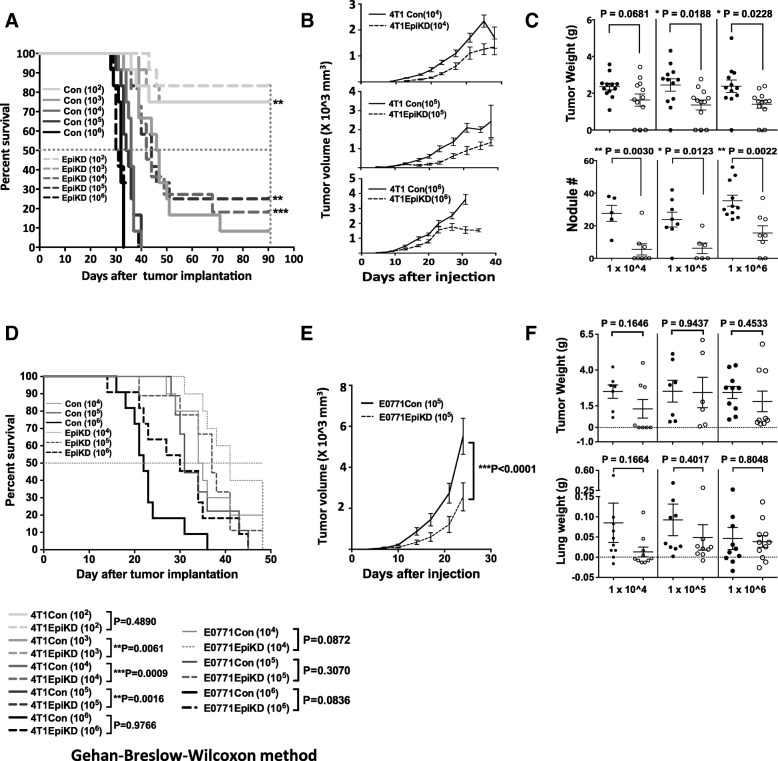
Prss14/ST14 is critical for metastasis of orthotopically-implanted 4 T1 and E0771 breast cancer cells. (**a**) Survival curves of host mice orthotopically injected with 4 T1 and 4 T1EpiKD cells. 1 × 10^2^, 1 × 10^3^, 1 × 10^4^, 1 × 10^5^, or 1 × 10^6^ of 4 T1 control (Con; solid lines) or 4 T1EpiKD (EpiKD; dotted lines) cells were implanted into the mammary fat pad of Balb/c mice. The cell numbers are indicated in the graph. (**b**) The growth curves of 4 T1Con and 4 T1EpiKD primary tumors. From top to bottom, 1 × 10^4^, 1 × 10^5^, and 1 × 10^6^ cells. (**c**) The weight of 4 T1 primary tumor and the numbers of 4 T1 lung metastatic nodules. Upper panels: primary tumor weight, lower panels: nodule numbers. Filled circle: 4 T1Con, empty circle: 4 T1EpiKD. Means and standard errors are indicated. (**d**) Survival rate of C57BL/6 mice implanted with E0771-Control-D11 (E0771Con, solid line) or E0771-EpiKD-C3 (E0771EpiKD, dotted line) cells (1 × 10^4^, 1 × 10^5^, 1 × 10^6^ cells/mouse (*n* = 10)). (**e**) The growth curves of E0771Con and E0771EpiKD primary tumors. (**f**) Comparison of the E0771 primary tumor weight and E0771 lung weight. Filled circle: E0771Con, empty circle: E0771EpiKD. *P* values measured by unpaired t test are shown

In order to assess the role of Prss14/ST14 on the breast cancer progression and metastasis, we used two mouse breast cancer cell lines, highly metastatic 4 T1 and less metastatic E0771, and their syngeneic mouse hosts, Balb/c and C57BL/6 strains, respectively (Fig.1). Prss14/ST14 knock down cells (EpiKD) and their control partner cells (Con) were orthotopically injected into mammary fat pads, and followed by the analysis of survival, tumor onset, growth of the primary tumor, and metastasis to the lung. The 4 T1 EpiKD [20] increased the survival in a dose dependent fashion (Fig. 1a) while slightly decreased in the growth rate of the primary tumor (Fig. 1b and c). Also, 4 T1 EpiKD significantly reduced the numbers of metastatic nodules in the lung in comparison to the orthotopically injected 4 T1 cells (Fig. 1c). Newly generated cell line E0771 EpiKD (Additional file 1: Figure S1) was used for the same kind of experiments (Fig.1d-f). Knock down of the Prss14/ST14 message in E0771 also slightly increased the survival rate (Fig. 1d). Tumor growth (Fig. 1e and f) and metastasis indicated the decreasing trend in lung weight (Fig. 1f), but the statistical significances were much lower than those for the results of 4 T1 EpiKD. In the case of 4 T1 breast cancer model, which shows greater metastatic nature than E0771, the metastasis of 4 T1 to the lung is also clearly suppressed by the knockdown. These results suggested that the Prss14/ST14 is required for efficient 4 T1 tumor progression and metastasis.

### Immunization of Prss14/ST14 antigenic peptides is effective in abrogating metastasis of 4 T1 breast cancer

**Fig. 2:**
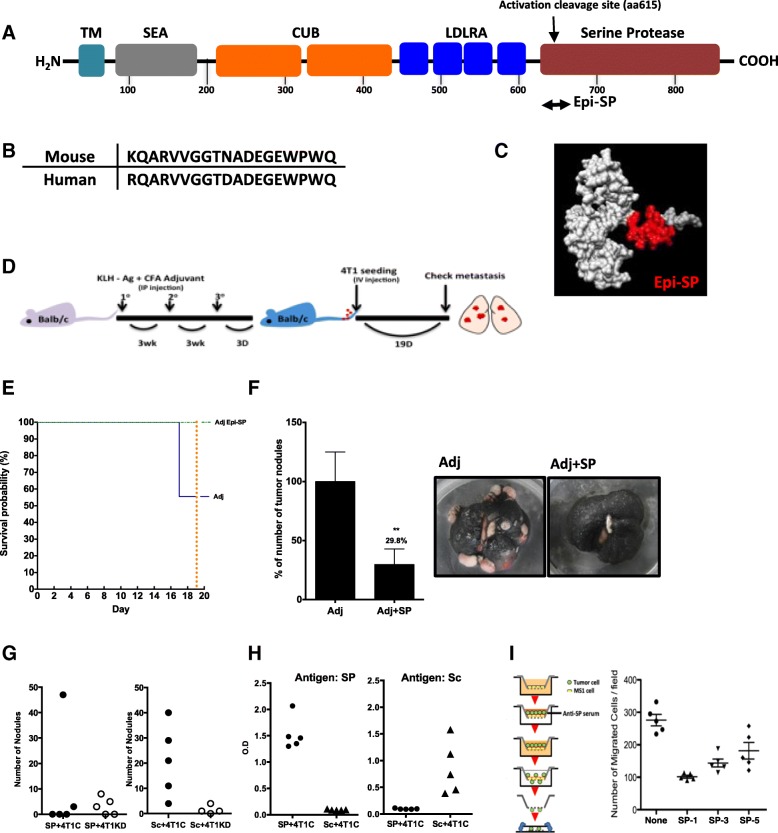
Conjugated peptide antigen as a preventive metastasis cancer vaccine. (**a**) Location of antigen Epi-SP in whole protein. (**b**) The sequences of mouse and human Epi-SP. (**c**) The Location of Epi-SP (red) in the structure model. (**d**) Immunization protocol. (**e**) The survival curves with immunization. Mice were sacrificed on day 19 to assess metastasis. (**f**) Number of metastasis nodule with immunization (Left). The representative images of metastasis (Right). (**g**) The levels of 4 T1 metastasis inhibition with immunization of Epi-SP are similar to the levels of 4 T1EpiKD. 4 T1C: 4 T1Con, 4 T1KD: 4 T1EpiKD. (**h**) Antibody specificity to Epi-SP and Epi-Sc. Antigens were immunized three times and antibodies were examined by ELISA. SP: EPi-SP, Sc: EPi-Sc. (**i**) Antiserum from the immunized mice blocks transendothelial migration. Overall scheme of the experiment is shown on the left, cells migrated through MS1 endothelial cells toward the opposite side are shown on the right. Average and standard error of means are shown

Since Prss14/ST14 plays the critical role of activating multiple downstream substrates, we made an assumption that inhibiting function with antibodies can block tumor metastasis and increase survival of tumor patients. Therefore, we decided to design the antigenic epitopes that reveal high antigenicity, hydrophilicity, surface probability, evolutionary conservation, and avoided the area of protein modification such as glycosylation. The most interesting candidate initially selected from the region was the activation loop of the protease domain Epi-SP (19mer) (Fig. 2a). This sequence includes the activation cleavage site (QARVVG) and is highly conserved between mouse and human (Fig. 2b). These epitopes are located in the appropriate positions to be antigens in the models (Fig. 2c). Therefore, we decided to test it as a preventive anti-metastasis vaccine. Immunization of the KLH conjugated mouse epitope peptide, Epi-SP, produced easily detectible antibodies in Balb/c mouse.

In order to test the possibility of reducing cancer metastasis, the metastasis assay by tail vein injection was applied after three immunizations in complete Freund’s adjuvant and incomplete Freund’s adjuvant (Fig. 2d). At the time point that mice were sacrificed, metastatic nodules on the lungs were counted (Fig. 2e, f). Epi-SP caused a statistically significant reduction in the numbers of metastasis nodules, indicating that immunizing cancer self Prss14/ST14 antigens can interfere with cancer metastasis.

To exclude the possibility of nonspecific effects for blocking metastasis by raised antibodies, Epi-SP sequence-scrambled peptide, Epi-Sc was selected (Fig. 2g and h). When two antigens were tested in parallel with the 4 T1 and 4 T1 EpiKD cells for the tail vein metastasis assays with immunization, 4 out of 5 mice immunized with the Epi-SP peptide exhibited clear abolition of metastasis, while Epi-Sc failed to block metastasis (Fig. 2g). The levels of reduction in metastasis by immunization were similar to the levels of 4 T1 EpiKD cells. These data showed that the Prss14/ST14 peptide reduces 4 T1 cancer metastasis in a sequence specific manner. Each antiserum only bound to their specific antigen sequence with high specificity (Fig. 2h), indicating there is no cross reactivity between two antibodies.

To address the question of how the immunizing Epi-SP peptide can reduce 4 T1 cancer metastasis, abilities of transendothelial migration in the presence of mouse sera were examined in vitro. 4 T1 cancer cells seeded onto the confluent monolayer of MS1 endothelial cells migrated less in the presence of the anti-Epi-SP sera (Fig. 2i). This suggested that the antibodies induced with Epi-SP peptide interfered with metastasis through blocking transendothelial migration of the cancer cells.

### Stable autocatalytic circular loop peptides can interfere with E0771 metastasis efficiently

**Fig. 3:**
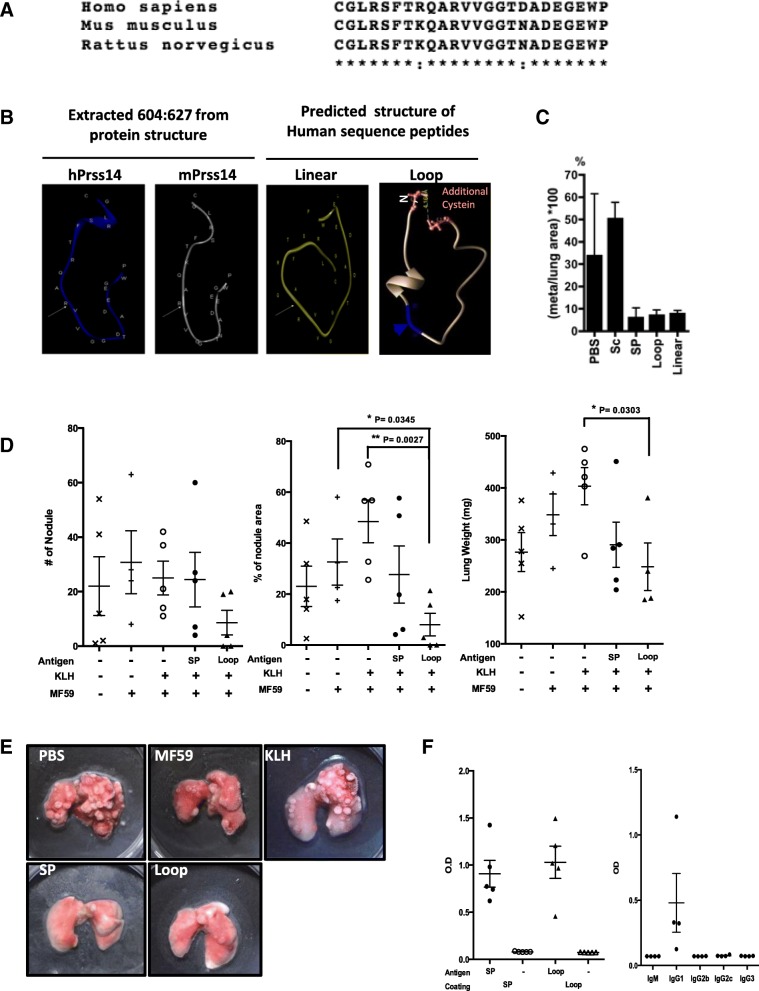
Circular autoactivation loop antigen modified to form stable structure worked as a preventive metastasis vaccine in C57BL/6 mouse. (**a**) The amino acid sequence 604:627 of human Prss14/ST14 that contains an autoactivation cleavage site is well conserved. (**b**) The modeling of the aa604:627 structures from human and mouse Prss14/ST14 whole protein models (left two panels). aa604:627 peptide does not retain the original loop structure (Linear). A cysteine added to the 627th amino acid to form cysteine bond makes the loop structure (Loop). The activation cleavage site is indicated with arrows. (**c**) Preventive metavaccine effects of various peptide antigen forms. 1 × 10^6^ E0771 cells were injected via tail vein of C57BL/6 mice after immunization of EP-SP, Mat-Loop, and Mat-Linear with Alum. (metastatic area/ whole area *100). (**d**) Metastasis inhibition of SP, Loop peptides, and KLH carrier protein. E0771 cells (1 × 10^6^) were injected via tail vein after immunization with MF59. The lung weight was measured on the day of sacrifice. (E) The representative images of the metastasis. (**f**) The examination of antibodies (Left) and isotypes (Right)

We attempted to raise specific monoclonal antibodies against the Epi-SP sequence without success. These unsuccessful trials led us to think that the epitope peptide may not be stable enough for screening the high affinity antibodies. Thus, we reinvestigated the structure models by measuring the distance of the amino acids in order to make a stable loop (Additional file 1: Figure S2). The 24mer peptide spanning from aa604 to aa627 showed the closest ends covering the autoactivation loop region. This region is highly conserved in three species (Fig. 3a). There is only one amino acid difference between the human and two murine sequences. We added cysteine at the C terminal in order to make the disulfide bond by reduction (25mer). Structure modeling of the autoactivation loop based on the structure analyses of whole human and mouse protein derived from the crystal studies revealed very similar structures while modeling of circular 25mer peptide sequences were comparable (Fig. 3b).

The first immunization of three KLH conjugated peptides, mouse sequence derived Epi-SP and human derived linear 24mer (Mat-Linear) and cysteine bonded 25mer loop (Mat-Loop) with Alum adjuvant, showed significant reduction while scrambled Epi-Sc antigen did not in C57BL/6 metastasis assay (Fig. 3c). When KLH conjugated antigen was tested along with two adjuvants, Alum and MF59, it was clear that Mat-Loop could reduce the numbers and total areas of the metastasis nodules of E0771 cells in the lung (Fig. 3d, e). In addition, the size and weight increase of the lung in a highly efficient manner that exceeds adjuvant effects with statistical significance. It appeared that antibodies generated in these conditions were all IgG1 isotypes, suggesting that the T_H_2 type response is dominating (Fig. 3f).

### mAb3F3 shows specificity to human protein and cross-reactivity to mouse Prss14/ST14 loop

**Fig. 4:**
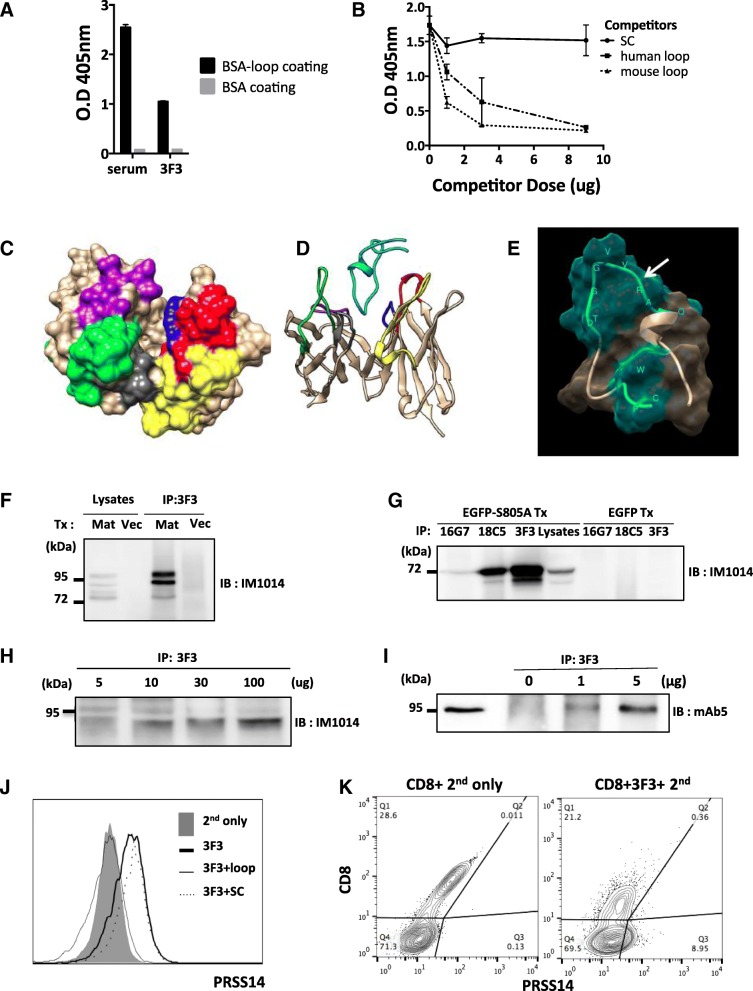
Production and characterization of mAb specific to autocatalytic loop. (**a**) mAb3F3 specifically binds to the autocatalytic loop. ELISA of mAb3F3 binding to the BSA conjugated Mat-Loop, and not to BSA only. (**b**) Competition human (Mat-Loop) and mouse loop (Epi-Loop) peptides to mAb3F3 binding on ELISA, coated with BSA conjugated human loop peptide. SC, scrambled sequence did not compete. (**c**) Modeling of mAb3F3. Red: H-CDR1, Yellow: H-CDR2, Blue: H-CDR3, Green: L-CDR1, Purple: L-CDR2, Gray: L-CDR3. (**d**) Docking modeling of mAb3F3 to Mat-loop peptides (lowest energy, − 238). Cyan: loop peptide. (**e**) Amino acids interacting with mAb3F3 in Mat-loop peptide were indicated as cyan. White arrow indicates the activation cleavage site. (**f**-**i**) Immunoprecipitation and western blotting for testing specific interaction of mAb3F3 to native Prss14/ST14 in cells. (**f**) Whole lysates of HEK293T cells transfected with full-length human Prss14 (Mat) and vector (Vec) were reacted with mAb3F3. (**g**) Whole lysates of HEK293T cells transfected with EGFP-S805A and EGFP was immunoprecipitated with mAb3F3 (**h**) Immunoprecipitation of MCF7. (**i**) Immunoprecipitation of 4 T1. (**j**) Flow cytometry of human Prss14/ST14 expressing HEK293T cells. Mat-Loop and Epi-Sc were used for competition. (**k**) Flow cytometry with CHO-S cells transiently transfected with CD8 or full length Prss14/ST14

From the successful metastasis blocking result obtained in the preventive metastasis vaccine using 25mer circular peptide, we developed the specific monoclonal antibody that can recognize the autoactivation loop structures to block activation and thus, block protease activity. In order to generate structure specific mAb directly against the autocatalytic loop of Prss14/ST14, we tried the hybridoma with circular Mat-Loop peptide as an antigen. After extensive screening, mAb3F3 was characterized with regard to its binding specificity (Fig. 4). mAb3F3 binds specifically to the Mat-Loop peptide sequence as shown in the ELISA coated with BSA conjugated Mat-Loop, but not with BSA alone (Fig. 4a). mAb3F3 binding to the BSA conjugated Mat-Loop on the ELISA plate was competed with Mat-Loop as well as Epi-Loop (Fig. 4b), revealing the cross reactivity to human and mouse protein sequences. The specific inhibitions were also tested in ELISA with coating peptide antigen through the C-terminal end (data not shown).

Sequencing mRNA from the hybridoma revealed CDR1,2,3 regions forming antigen binding pockets in both heavy and light chains (Table 1). Thereafter, the structure of mAb3F3 Fab region was predicted based on the amino acid sequences (Fig. 4c) and applied in docking modeling (Fig. 4d). As shown, the antigenic loop, taken from the whole protein model, fits nicely to the antibody binding pocket. The CDRs of mAb3F3 covers the activation cleavage site (Fig. 4e). SPR sensorgrams determined affinity of mAb3F3 against BSA conjugated peptide antigens (Additional file 1: Figure S3). The mAb3F3 affinities against both human loop (Mat-Loop) and mouse loop (Epi-Loop) peptides were at the nanomole levels (human: K_D_ = 5.333 × 10^− 9^, mouse: K_D_ = 7.814 × 10^− 9^) (Table 2). The linear form showed two logs lower affinity while the scrambled sequence showed no binding at all.

**Table 1:** Amino acid sequences of complementarity-determining region in mAb3F3

	Light Chain Variable Region (V_L_)	Heavy Chain Variable Region (V_H_)
Total Length	112	113
Sequences of CDR1	RSSQSIVHSNGNTFLE	GYTFSIYWLE
Sequences of CDR2	KVSNRFS	EILPGSGNANYNEKFKG
Sequences of CDR3	FQGSHVPFT	SGTD

**Table 2:** Kinetic measurement of mAb3F3 against various peptides antigen using SPR

	ka(1/Ms)	kd(1/s)	K_D_(M)
Mat-loop-BSA	6.414 × 10^5^	0.003420	5.333 × 10^−9^
Epi-loop-BSA	4.131 × 10^5^	0.003228	7.814 × 10^−9^
Mat-Linear	2.593 × 10^4^	0.01052	4.057 × 10^−7^
Scrambled	No binding		

### mAb3F3 shows the specificity to the native form of Prss14/ST14 protein present in human and mouse breast cancer cells

When the peptide antigen was used to raise monoclonal antibody, an important issue was to see if mAb3F3 could recognize the native protein structure. Therefore, we tested whether mAb3F3 binds to Prss14/ST14 expressed in cells. HEK293T cells were transfected with full length human Prss14/ST14 (Mat) and tested by immunoprecipitation (Fig. 4f and g). As shown in the figures, simple western blotting revealed four major different processed forms when the full length construct was transfected into HEK293T cells, mAb3F3 strongly immunoprecipitated two longer bands, unprocessed and processed at aa149 (Fig. 4f). When a construct consisting of recombinant EGFP fused with Prss14/ST14 protein containing S805 mutation, EGFP-S805A, was transfected, it was found in the immunocomplex with mAb3F3 (Fig. 4g). The control transfection with EGFP did not show any detectible band. Then we verified that mAb3F3 bound to endogenous Prss14/ST14 expressed in human MCF7 (Fig. 4h). mAb3F3 can also easily detect mouse protein of 4 T1 breast cancer cells (Fig. 4i). These results suggested that mAb3F3 specifically binds to the mouse and human native proteins. To further verify that mAb3F3 can bind to native Prss14/ST14 in another assay, we used flow cytometry (Fig. 4j and k). mAb3F3 was able to detect Prss14/ST14 protein expressed in the cells in a sequence specific competitive manner. Sequence specificity was apparent since binding can be competed away with only the peptide of its own, not of the scrambled. Binding specificity was also verified using two dimensional analysis with CD8 protein expressing cells as a negative population.

### mAb3F3 can detect PRSS14/ST14 expressed in various cancer cell lines

**Fig. 5:**
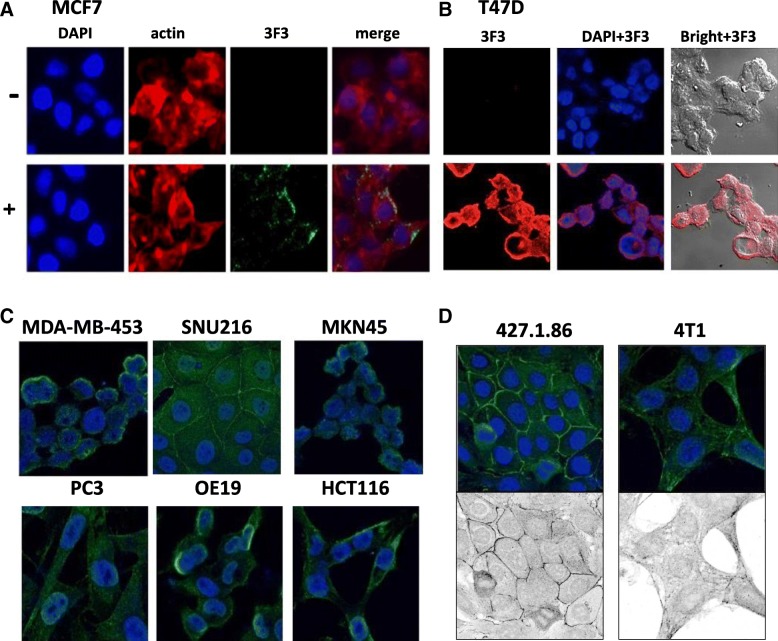
Immunofluorescence detection of Prss14/ST14 expression in various human and mouse cancer cells. (**a**) MCF7 breast cancer cell stained with DAPI, actin, and mAb3F3 (**b**) T47D breast cancer cell stained with mAb3F3 and DAPI. (**c**) Various cancer cells stained with mAb3F3. MDA-MB-453: human triple negative breast cancer cell line, SNU216: human gastric adenocarcinoma cell line, MKN45: human gastric cancer cell line, PC3: human prostate cancer cell line, OE19: esophageal adenocarcinoma cell line, HCT116: human colon carcinoma, (**d**) 427.1.86: mouse thymoma cell line, 4 T1: mouse breast cancer cell line. Bottom, Photoshop stylized presentation of staining pattern

We also investigated the possible usage of mAb3F3 as a diagnostic tool. mAb3F3 was applied in immunocytochemical staining on various cancer cell lines (Fig. 5). Specific Prss14/ST14 staining in the cytoplasm, cell contacts, and the membrane were detected in human breast cancer cells, MCF7 and T47D (Fig. 5a and b). There was no apparent background staining of the secondary reagent, and nuclear staining was found. Interestingly, Prss14/ST14 staining was found only in the subpopulation of MCF7 cells (Fig. 5a). An extended application on other human and mouse cancer cell lines with expression of Prss14/ST14 showed mAb3F3 can stain membrane or cell contact areas of MDA-MB-453 triple negative breast cancer, SNU216 and MKN45 human gastric adenocarcinoma, PC3 human prostate cancer, OE19 human esophageal adenocarcinoma, and HCT116 human colon carcinoma (Fig. 5c). In SNU216 cells, staining was also clearly present in the cytoplasm. 427.1.86 mouse thymoma cell and 4 T1 mouse breast cancer cell lines were also stained very well (Fig. 5d). All the cell lines used here were known to express Prss14/ST14 protein. Therefore, mAb3F3 can be a new unique diagnostic reagent to detect an unactivated form of Prss14/ST14 protein with the intact uncleaved activation loop structure.

### mAb3F3 decreases cancer cell migration and metastasis but does not effect on cell growth or cell death

**Fig. 6:**
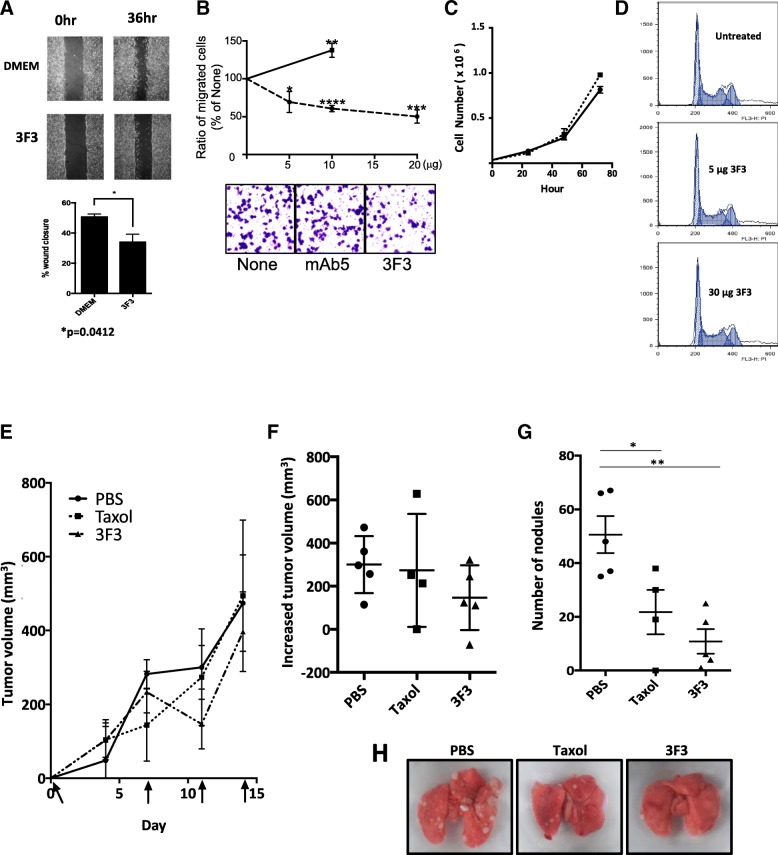
mAb3F3 can reduce cell migration and metastasis of MMTV-PyMT breast cancer. (**a**) Wound healing migration assay with 4 T1. mAb3F3 culture supernatant was added to the final concentration of 10%. (**b**) Transwell migration assay with MCF7. Dose response curves and representative fields are at the bottom. mAb5: Solid line, mAb3F3: Dotted line. (**c**) 5 × 10^4^ cells of MCF7 were seeded in 12 well plates. After 12 h, MCF7 were treated with mAb3F3 or control. Number of cells were checked every 24 h. (**d**) Cell cycle analysis by Propidium Iodide staining. Same numbers of MCF7 cells were seeded. (**e**) Growth curves of average tumor sizes from PBS, Taxol, and mAb3F3 injected PyMT mice. Arrows indicate injection time points (days) of mAb3F3. (**f**) Tumor volumes of each group at day11. (**g**) Number of metastasis nodules at day14. (**h**) Representative lung images of each group at the bottom

The roles of mAb3F3 were first tested in vitro. Addition of mAb3F3 hybridoma culture supernatant to 4 T1 culture reduced cell migration induced by wound healing in a statistically significant way (Fig. 6a). Addition of purified mAb3F3 to MCF7 culture reduced cell migration induced by serum gradient through transwell in a dose dependent manner (Fig. 6b). In contrast, mAb5 that binds to the denatured Prss14/ST14 did not reduce the migration. Purified mAb3F3 did not affect MCF7 cell growth (Fig. 6c) nor cell death when tested by cell cycle analysis (Fig. 6d).

Next, tumor bearing MMTV-PyMT mice were treated with mAb3F3 antibody or with taxol from 6 weeks of age (day 0). There were no significant differences found in the increase of primary tumor volume among the three groups except small size decreases in the mAb3F3 treated mice at day 11 (Fig. 6e and f). However, metastasis in the lungs revealed significant reduction of the number of tumor nodules at day 14 (Fig. 6g and h). These results indicated that mAb3F3 can inhibit cancer metastasis in the MMTV-PyMT mouse model, but not tumor growth with our protocol applied. From these results, we concluded that mAb3F3 is a promising candidate of preventive/therapeutic antibody to target metastasis.

### Humanized mAb3F3 maintained specificity and reduced cancer cell migration

In order to humanize mAb3F3, we engineered huAb3F3 by grafting murine CDRs onto its similar human germline sequences (Additional file 1: Fig. 4). Prior to undertaking the humanization of mAb3F3, a chimeric antibody (ch3F3) was also constructed in which the entire mouse mAb3F3 variable region was attached to human constant regions. Purified antibody preparation located in a main peak close to 100% of the total integrated peak area as determined by size exclusion HPLC without significant aggregation (data not shown). Humanized antibodies were characterized for binding affinity using SPR-based assay (Additional file 1: Figure S5). The binding affinity of ch3F3, two humanized clones, huAb3F3–35 and huAb3F3–37 measured were in the sub-nanomolar range.

**Fig. 7:**
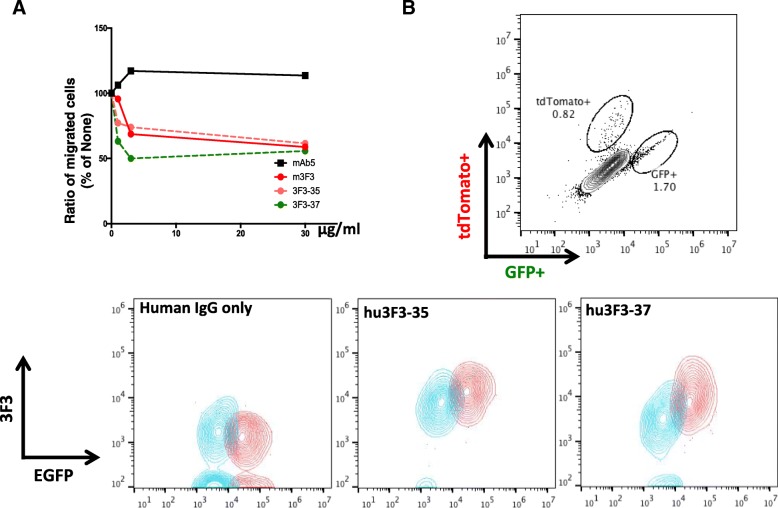
huAb3F3 clones inhibit cell migration and specifically bind to uncleaved protein. (**a**) Transwell migration assay with MCF7. 5 × 10^4^ cells were seeded on the upper chamber and incubated with antibody without serum. After 4 h, the lower chamber was filled with media with serum for 24 h. mAb5: Solid black line, m3F3 (mAb3F3): Solid red line, 3F3–35 (huAb3F3–35): Dotted orange line, 3F3–37 (huAb3F3–37): Dotted green line. (**b**) Flow cytometric analysis of HEK293TF cells co-transfected with either EGFP and matriptaseS805A or with tdTomato using huAb3F3 Ab clones. Blue population: tdTomato^+^, Red population: GFP^+^

Using two humanized clones huAb3F3–35 and huAb3F3–37, we tested their abilities in the MCF7 cell migration through transwell (Fig. 7a). The levels of cell migration inhibition were clearly comparable to the parent mAb3F3 (huAb3F3–35) or even better (huAb3F3–37) in dose dependent fashion. A negative control monoclonal antibody, mAb5, that recognized denatured protease domain did not reduce transwell migration.

To verify the specificity of the humanized clones, we applied flow cytometry using the HEK293TF cell line by transfecting a protease activity mutant, MatS805A along with EGFP. Negative population was transfected only with tdTomato. As seen in Fig. 7b, huAb3F3–37 showed distinctly better differences in the levels of staining with MatS805A cells, suggesting cleaner specificity than huAb3F3–35 (Fig. 7b).

These results suggested that humanized antibody huAb3F3–37 is a strong candidate to apply as a reagent targeting breast cancer diagnosis and/or as a therapeutic reagent.

## Discussion

In this study, we showed that Prss14/ST14 is a critical marker for breast cancer metastasis and a therapeutic target. Structural epitope peptide can prevent metastasis when immunized prior to injection of metastatic breast cancer and mAb directed to the activation loop of the protein can detect the native protein and inhibit metastasis.

As we are currently gathering more information on the mechanism of metastasis, it now appears as a complicated multistage and multipathway process [36, 37]. Either as a single cell dissemination or a collective tumor cell migration, most tumor cells undergo EMT and extravasation process after escaping hypoxia by angiogenesis to settle at distant sites. In addition, heterogeneous cancer cells can escape from therapy to migrate out of its original site. For targeting breast cancers, recent reviews summarized the components and suggested key pathways [38, 39]. We are confident that this research will be a great addition to the list.

### Prss14 as a therapeutic target against metastatic breast cancer

We showed Prss14/ST14 is a strong prognosis marker for highly mortal ER^−^ breast cancer patients [8]. The survival of ER^−^ breast cancer patients with high expression level of Prss14/ST14 is extremely poor while no death was apparent with patients with low expression level. Prss14/ST14 is valuable as a marker for poor survival of the post-surgery esophageal cancer [40]. Using several immunocompetent models, we verified that the importance of Prss14/ST14 roles in metastasis in breast cancer (Figs. 1, 2, 3 and 6). The survival of mice orthotopically transplanted with 4 T1 or E0771 breast cancer cells were significantly increased when Prss14/ST14 was knocked-down. Prss14/ST14 functions have more effect in metastasis than in the growth of the primary tumors.

Despite the high mortality of patients with malignant tumors, there are no active preventive strategy on future metastasis after the primary tumor mass has been removed. Either dietary or adjuvant therapy are currently the most applicable options, probably because there is no usable target for blocking metastasis. There has been an earlier attempt to reduce metastasis using CD44 [41], uPA [42], or AECHL-1 as targets [43]. Now, we strongly believe that Prss14/ST14 is as good as, if not better, a candidate for modulating metastasis. Prss14/ST14 is involved in all the important processes of metastasis, angiogenesis, EMT, cell invasion into matrix, cell migration, and transendothelial migration since it digests matrix proteins and activates many metastatic pathways. More importantly, it can activate itself like a master switch. If we block the function of Prss14 as an active protease at the level of activation, we will be able to block the entire downstream events.

### Epitopes can behave as a preventive metastasis vaccine

To our surprise, the highly conserved autoactivation loop sequence (SP) and the modified peptide to maintain the loop structure to form a disulfide bond (Loop) (Fig. 2 and Additional file 1: Figure S2) behaved as efficient epitopes (Figs. 2, 3 and 6). The KLH conjugated epitopes with various adjuvants worked as preventive metastasis vaccines in two mouse metastatic breast cancer models (Fig. 2 and Fig. 3). In contrast to our initial concern of Prss14/ST14 being self-antigen, present in thymic epithelium, which induces self-tolerance [44, 45], all the mice generated sufficiently detectible Th2 type antibodies (Fig. 2h and Fig. 3f). Epi-SP, Mat-Linear, and Mat-Loop epitopes showed potential to be preventive metastasis vaccines (Fig. 2 and Fig. 3). They were able to raise high titer antibodies and eliminated metastasis nodules. Prevention of metastasis with immunization of these antigens was sequence specific. The reduction of metastasis was as efficient as the knocking down of Prss14/ST14, and better than the sole effect of the adjuvant. The mechanism of prevention of metastasis is at least in part by reducing the ability of transendothelial cell migration as shown in Fig. 2I. However, more detail mode of actions will require extensive studies.

### mAb3F3 as a new immunologic agent targeting metastasis

mAb3F3 that recognize the autoactivation loop of Prss14/ST14 was investigated for its specificity by several assays (Fig. 4). In ELISA, mAb3F3 specifically binds to the epitopes, either conjugated with BSA or C-terminal linked peptide which can maintain the structure of peptide. mAb3F3 clearly recognized the native forms of Prss14/ST14 expressed in cells detected by immunoprecipitation and by flow cytometry. The bindings to the native protein structure were competed with only the same sequences. The affinities to the human and mouse epitopes are within the range of nanomoles (Table. 2 and Additional file 1: Figure S3).

Because epitope structure is destroyed by cleavage within the autoactivation loop sequence, mAb3F3 can only bind the inactive form. It is going to be useful to distinguish the inactive form from the activated form. mAb3F3 can stain various cultured cancer cells (Fig. 5), suggesting its value as a diagnostic tool as well.

mAb3F3 clearly reduced in vitro cell migration, transwell assays, and metastasis in MMTV-PyMT mice while it did not interfere with cell growth, cell death, or cell cycle (Fig. 6). The effects of mAb3F3 on tumor metastasis were better than taxol treatment, suggesting that a combination of taxol and mAb3F3 may yield better inhibition. One humanized antibody huAb3F3–37 maintained a high level of specificity and as good as, if not better, in vitro effect on cell migration (Fig. 7a). Therefore, mAb3F3 and huAb3F3–37 are good candidates of therapeutic antibodies to the metastatic breast cancers and can be used as a component in combination therapy. If antibody mediated endocytosis is sufficient, these reagents can also be good lead materials as antibody drug conjugates for targeting the removal of early cancer cells with unactivated Prss14/ST14 protease on the surface.

## Conclusions

Our study demonstrates that Prss14/ST14 is an important target for metastasis modulation, an epitope derived from the structure, specifically the autoactivation loop, can be useful as a preventive a metastasis vaccine. Our newly developed mAb3F3 and huAb3F3 can recognize the structure and sequence specific epitope of the autoactivation loop, and may function as a therapeutic antibody targeting metastasis modulation.

## Additional files

Additional file 1: **Figure S1.** Prss14/ST14 knockdown in E0771 cell line. **Figure S2.** Designing antigen to maintain stable autoactivation loop structure. **Figure S3.** SPR sensorgrams show binding of human and mouse loop to mAb3F3. **Figure S4.** Amino acid sequence of humanized antibodies aligned with human germlines and mouse antibody. **Figure S5.** huAb3F3 antibodies showed a similar level of antigen binding capacity as the chimeric antibody. (PPTX 1022 kb)

## Data Availability

The authors declare that all data in this study are available in the article and additional files.

## Abbreviations

BSA: Bovine serum albumin
DMEM: Dulbecco’s Modified Eagle’s Media
ELISA: Enzyme-Linked Immunosorbent Assay
EMT: Epithelial to mesenchymal transition
Epi: Epithin
EpiKD: Epithin knockdown
Fab: Fragment antigen-binding
FBS: Fetal bovine serum
HAT: Hypoxanthine, Aminopterin, and Thymidine
HRP: Horseradish peroxidase
HT: Hypoxanthine and Thymidine
KLH: Keyhole limpet hemocyanin
mAb: Monoclonal antibody
Mat: Matriptase
MMTV: Mouse mammary tumor virus
MT-SP1: Membrane types serine protease 1
PEG: Polyethylene glycol
PI: Propidium Iodide
Prss14: Serine protease 14
Sc: Scrambled
SE-HPLC: Size-exclusion high-performance liquid chromatography
ST14: Suppression of tumorigenicity 14
TN: Triple negative
VH: Heavy chain variable region
VL: Light chain variable region
