# Supplementary material for: Targeting metastatic breast cancer with peptide epitopes derived from autocatalytic loop of Prss14/ST14 membrane serine protease and with monoclonal antibodies
Source: J Exp Clin Cancer Res. 2019 Aug 19;38:363. doi: 10.1186/s13046-019-1373-y (PMC6701106; doi:10.1186/s13046-019-1373-y)
Supplement: Supplementary file 1 — Figure S1. Prss14/ST14 knockdown in E0771 cell line. Figure S2. Designing antigen to maintain stable autoactivation loop structure. Figure S3. SPR sensorgrams show binding of human and mouse loop to mAb3F3. Figure S4. Amino acid sequence of humanized antibodies aligned with human germlines and mouse antibody. Figure S5. huAb3F3 antibodies showed a similar level of antigen binding capacity as the chimeric antibody. (PPTX 1022 kb) [file 13046_2019_1373_MOESM1_ESM.pptx]

## Slide 1
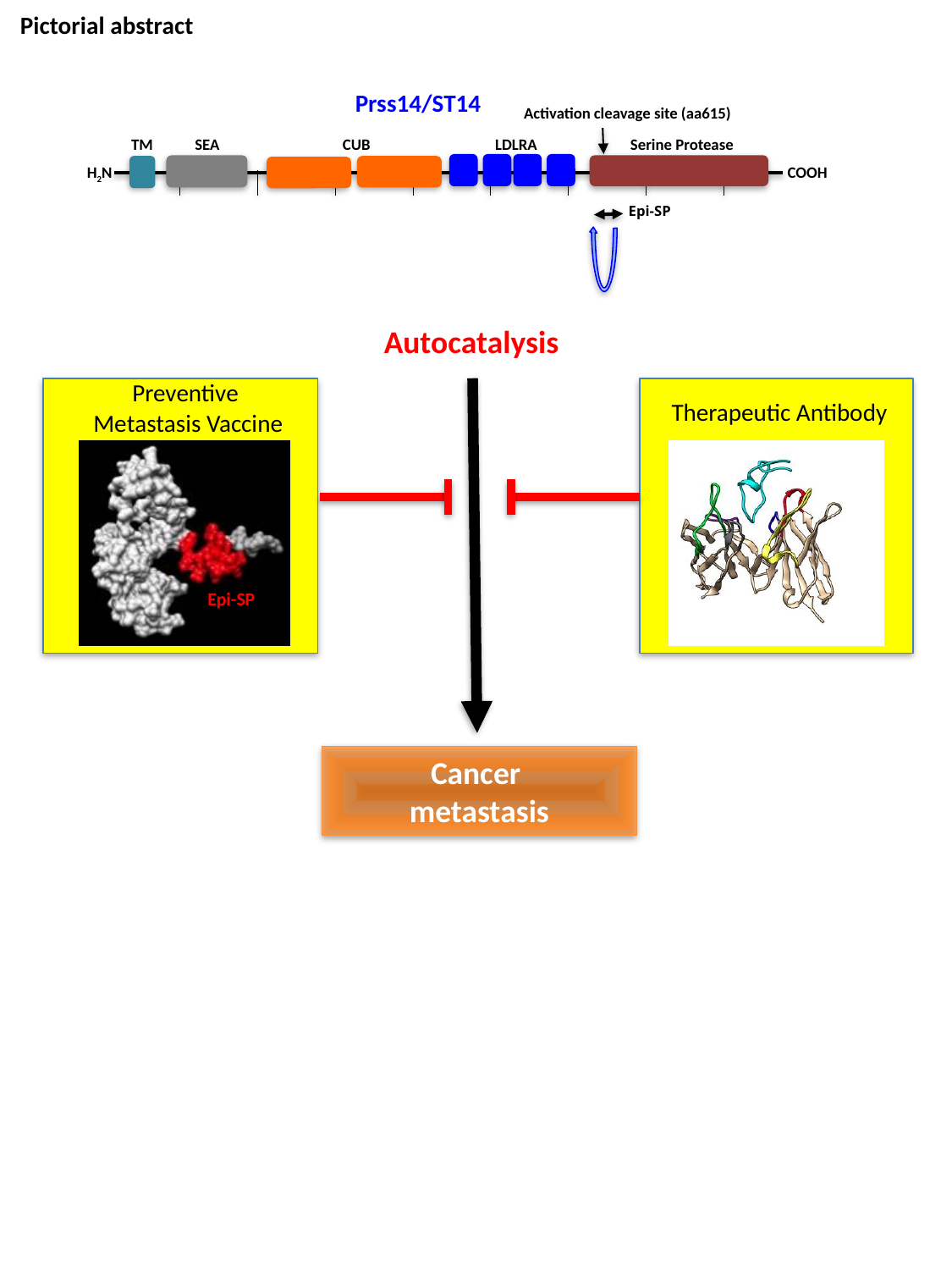

Pictorial abstract
Prss14/ST14
Activation cleavage site (aa615)
TM
SEA
CUB
LDLRA
Serine Protease
COOH
H2N
Epi-SP
Autocatalysis
Preventive
Metastasis Vaccine
Therapeutic Antibody
Epi-SP
Cancer
metastasis

## Slide 2
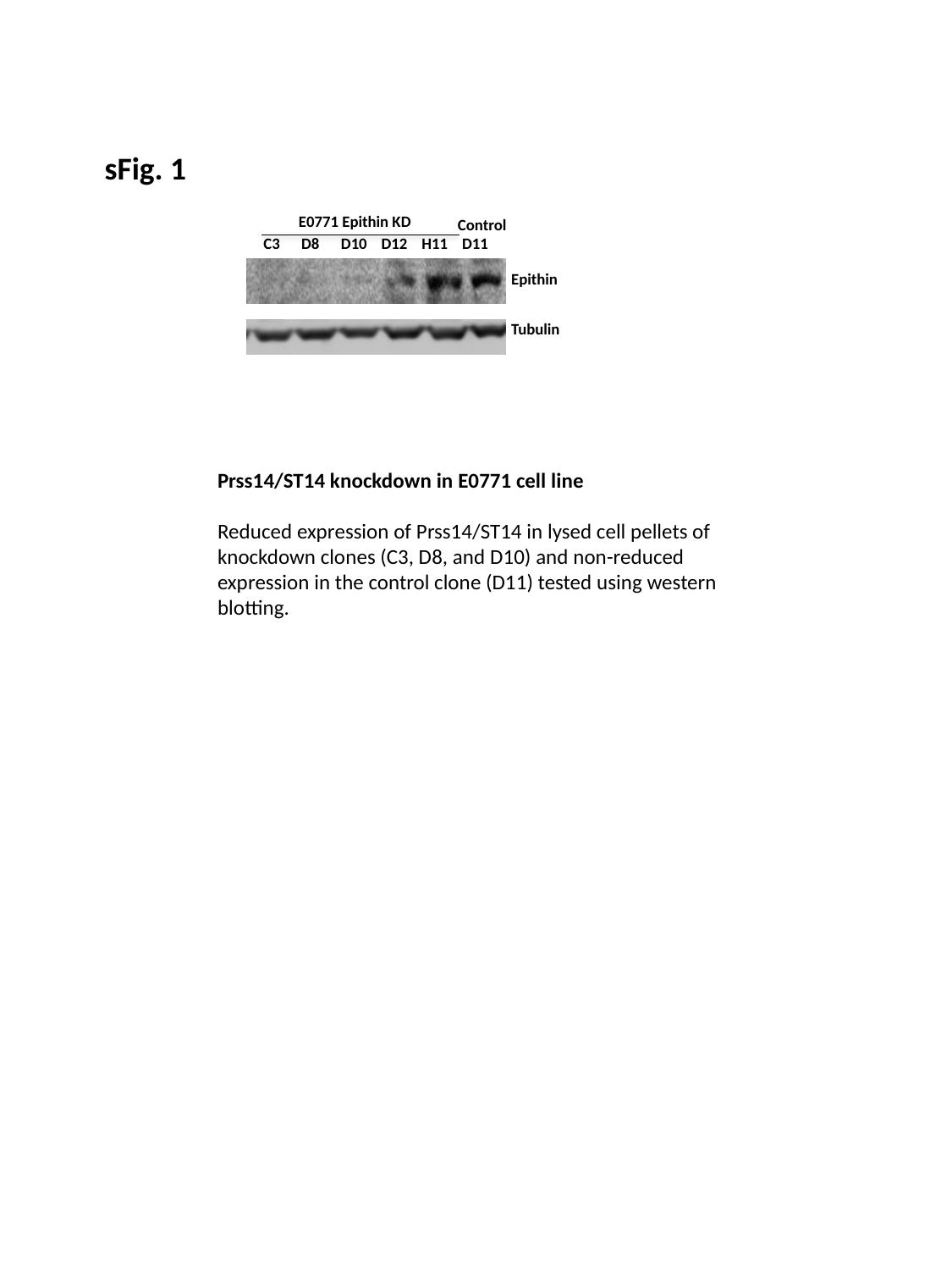

sFig. 1
E0771 Epithin KD
Control
C3 D8 D10 D12 H11 D11
Epithin
Tubulin
Prss14/ST14 knockdown in E0771 cell line
Reduced expression of Prss14/ST14 in lysed cell pellets of knockdown clones (C3, D8, and D10) and non-reduced expression in the control clone (D11) tested using western blotting.

## Slide 3
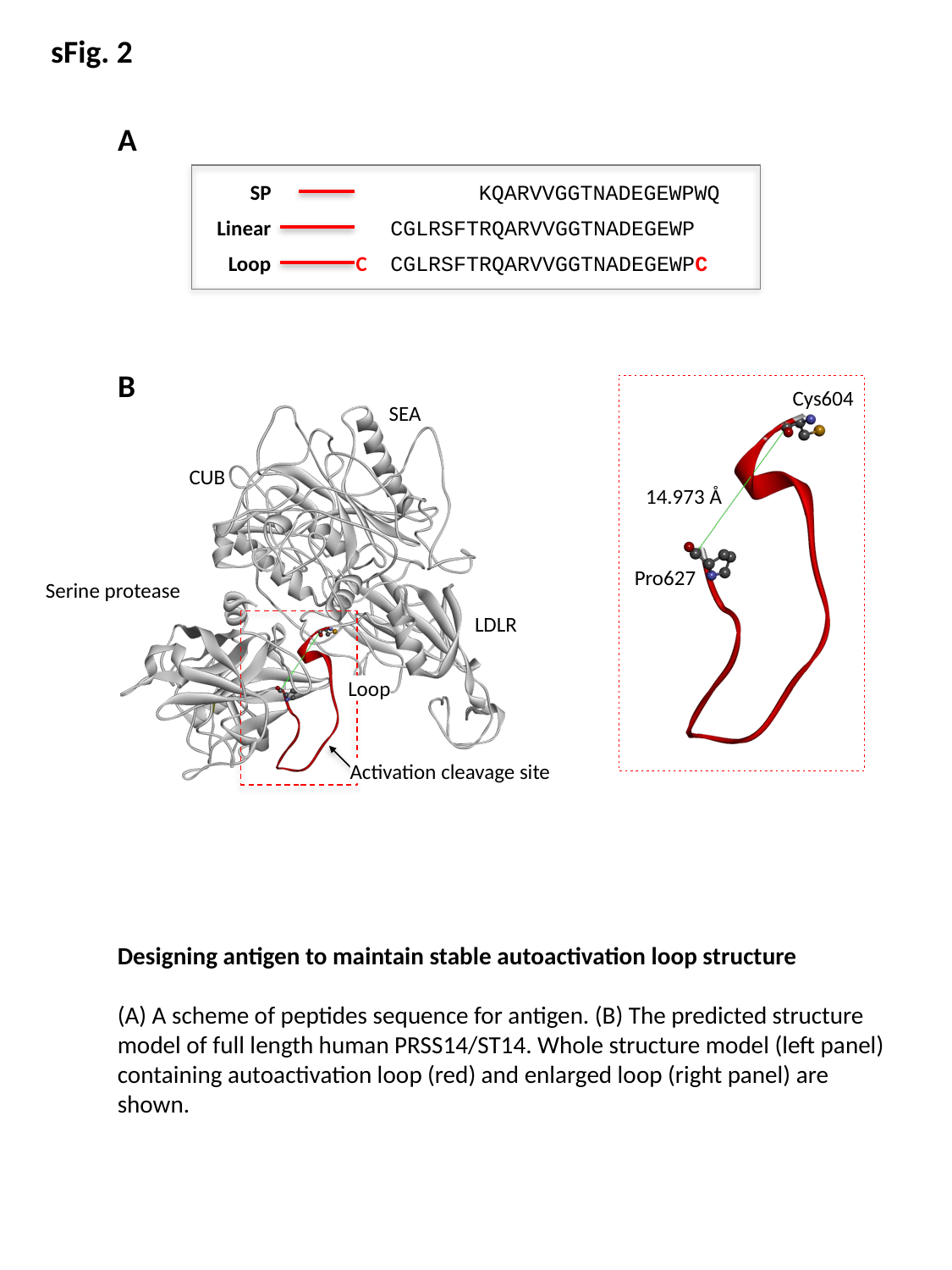

sFig. 2
A
SP
KQARVVGGTNADEGEWPWQ
Linear
CGLRSFTRQARVVGGTNADEGEWP
Loop
CGLRSFTRQARVVGGTNADEGEWPC
C
B
Cys604
SEA
CUB
14.973 Å
Pro627
Serine protease
LDLR
Loop
Activation cleavage site
Designing antigen to maintain stable autoactivation loop structure
(A) A scheme of peptides sequence for antigen. (B) The predicted structure model of full length human PRSS14/ST14. Whole structure model (left panel) containing autoactivation loop (red) and enlarged loop (right panel) are shown.

## Slide 4
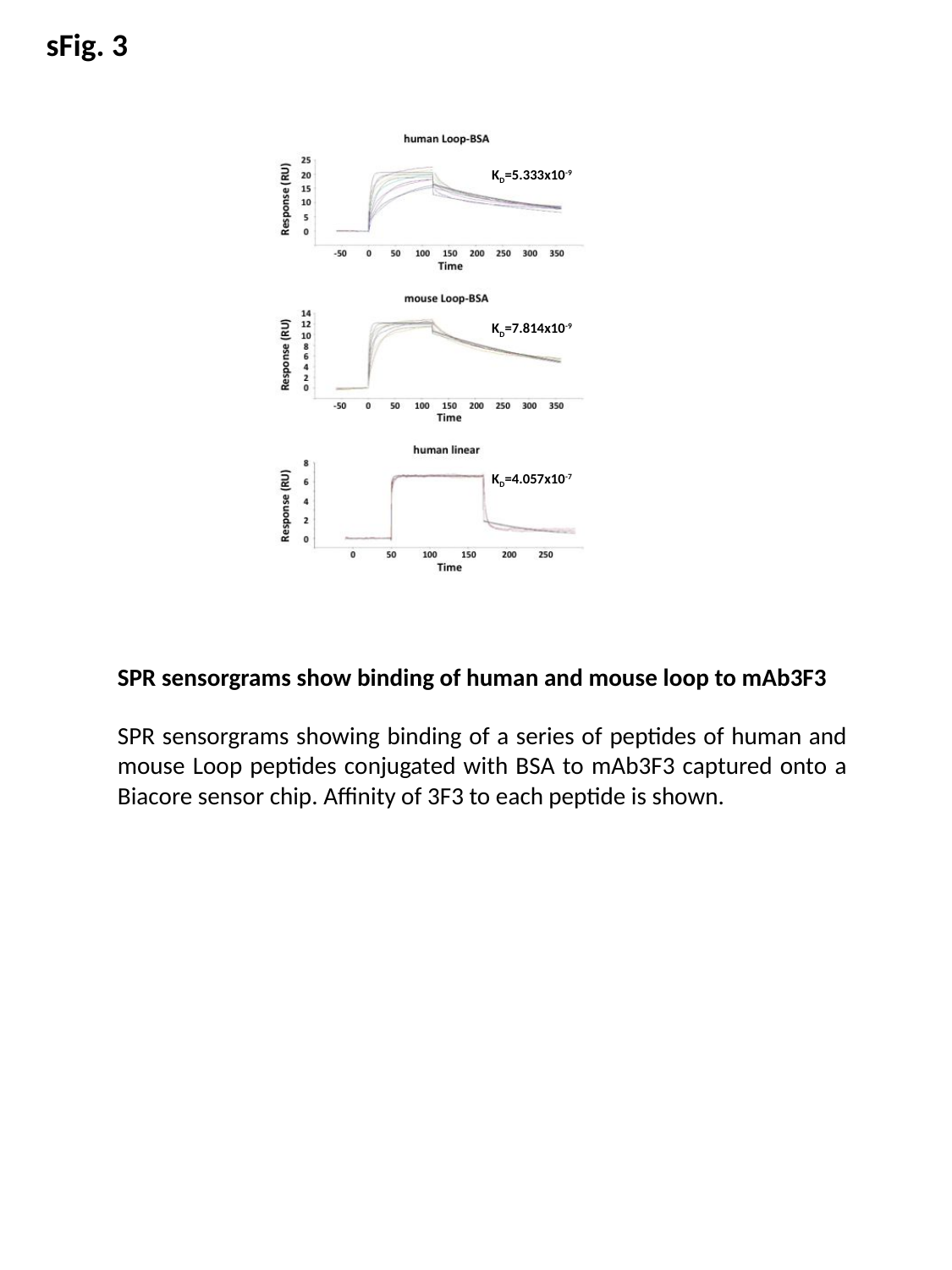

sFig. 3
KD=5.333x10^-9^
KD=7.814x10^-9^
KD=4.057x10^-7^
SPR sensorgrams show binding of human and mouse loop to mAb3F3
SPR sensorgrams showing binding of a series of peptides of human and mouse Loop peptides conjugated with BSA to mAb3F3 captured onto a Biacore sensor chip. Affinity of 3F3 to each peptide is shown.

## Slide 5
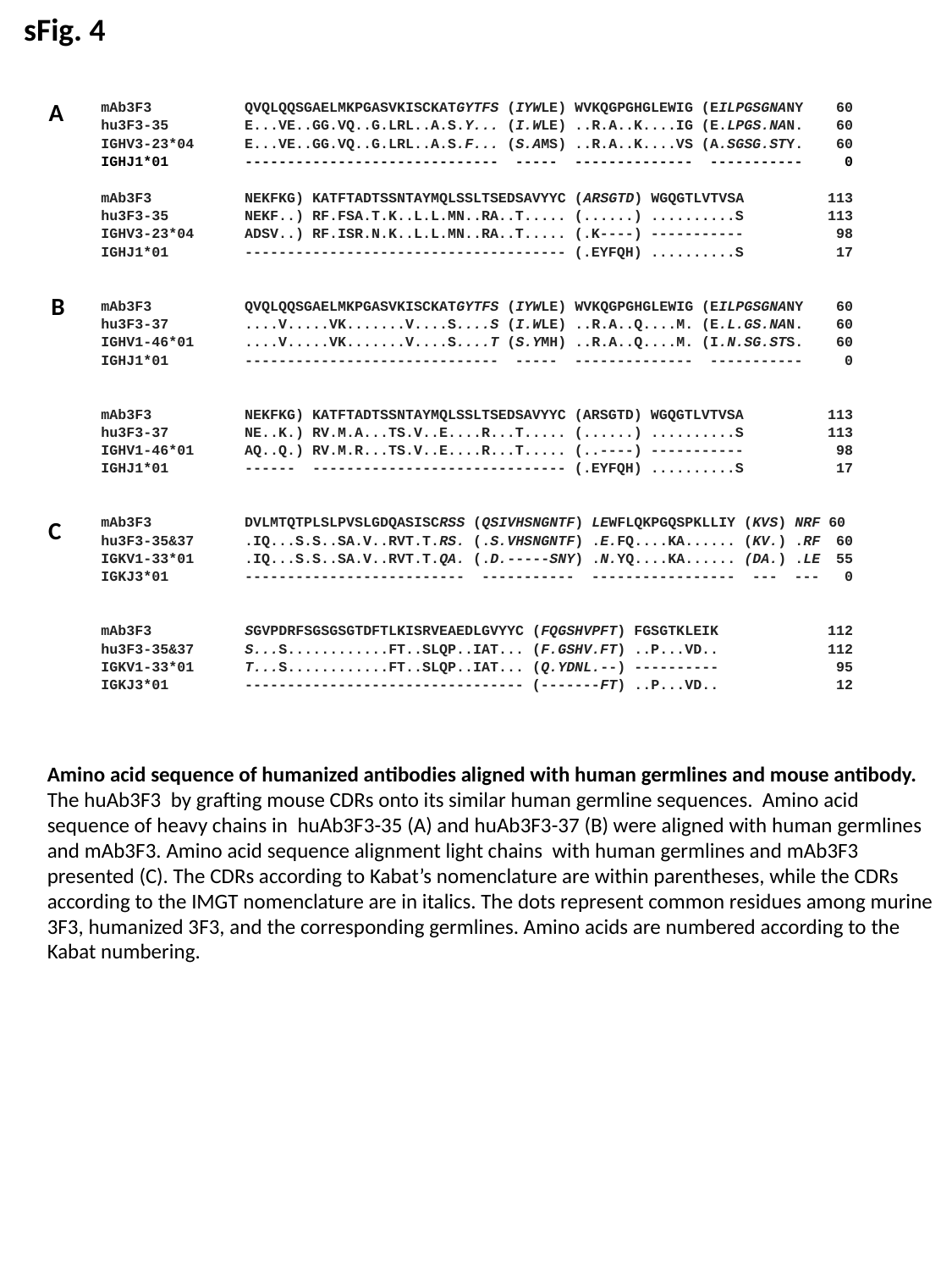

sFig. 4
A
mAb3F3 QVQLQQSGAELMKPGASVKISCKATGYTFS (IYWLE) WVKQGPGHGLEWIG (EILPGSGNANY	 60
hu3F3-35 E...VE..GG.VQ..G.LRL..A.S.Y... (I.WLE) ..R.A..K....IG (E.LPGS.NAN.	 60
IGHV3-23*04 E...VE..GG.VQ..G.LRL..A.S.F... (S.AMS) ..R.A..K....VS (A.SGSG.STY.	 60
IGHJ1*01 ------------------------------ ----- -------------- -----------	 0
mAb3F3 NEKFKG) KATFTADTSSNTAYMQLSSLTSEDSAVYYC (ARSGTD) WGQGTLVTVSA		113
hu3F3-35 NEKF..) RF.FSA.T.K..L.L.MN..RA..T..... (......) ..........S 	113
IGHV3-23*04 ADSV..) RF.ISR.N.K..L.L.MN..RA..T..... (.K----) -----------		 98
IGHJ1*01 -------------------------------------- (.EYFQH) ..........S 	 17
mAb3F3 QVQLQQSGAELMKPGASVKISCKATGYTFS (IYWLE) WVKQGPGHGLEWIG (EILPGSGNANY	 60
hu3F3-37 ....V.....VK.......V....S....S (I.WLE) ..R.A..Q....M. (E.L.GS.NAN.	 60
IGHV1-46*01 ....V.....VK.......V....S....T (S.YMH) ..R.A..Q....M. (I.N.SG.STS.	 60
IGHJ1*01 ------------------------------ ----- -------------- -----------	 0
mAb3F3 NEKFKG) KATFTADTSSNTAYMQLSSLTSEDSAVYYC (ARSGTD) WGQGTLVTVSA		113
hu3F3-37 NE..K.) RV.M.A...TS.V..E....R...T..... (......) ..........S		113
IGHV1-46*01 AQ..Q.) RV.M.R...TS.V..E....R...T..... (..----) -----------		 98
IGHJ1*01 ------ ------------------------------ (.EYFQH) ..........S 	 17
mAb3F3 DVLMTQTPLSLPVSLGDQASISCRSS (QSIVHSNGNTF) LEWFLQKPGQSPKLLIY (KVS) NRF 60
hu3F3-35&37 .IQ...S.S..SA.V..RVT.T.RS. (.S.VHSNGNTF) .E.FQ....KA...... (KV.) .RF	 60
IGKV1-33*01 .IQ...S.S..SA.V..RVT.T.QA. (.D.-----SNY) .N.YQ....KA...... (DA.) .LE	 55
IGKJ3*01 -------------------------- ----------- ----------------- --- ---	 0
mAb3F3 SGVPDRFSGSGSGTDFTLKISRVEAEDLGVYYC (FQGSHVPFT) FGSGTKLEIK		112
hu3F3-35&37 S...S............FT..SLQP..IAT... (F.GSHV.FT) ..P...VD..		112
IGKV1-33*01 T...S............FT..SLQP..IAT... (Q.YDNL.--) ----------		 95
IGKJ3*01 --------------------------------- (-------FT) ..P...VD..		 12
B
C
Amino acid sequence of humanized antibodies aligned with human germlines and mouse antibody.
The huAb3F3 by grafting mouse CDRs onto its similar human germline sequences. Amino acid sequence of heavy chains in huAb3F3-35 (A) and huAb3F3-37 (B) were aligned with human germlines and mAb3F3. Amino acid sequence alignment light chains with human germlines and mAb3F3 presented (C). The CDRs according to Kabat’s nomenclature are within parentheses, while the CDRs according to the IMGT nomenclature are in italics. The dots represent common residues among murine 3F3, humanized 3F3, and the corresponding germlines. Amino acids are numbered according to the Kabat numbering.

## Slide 6
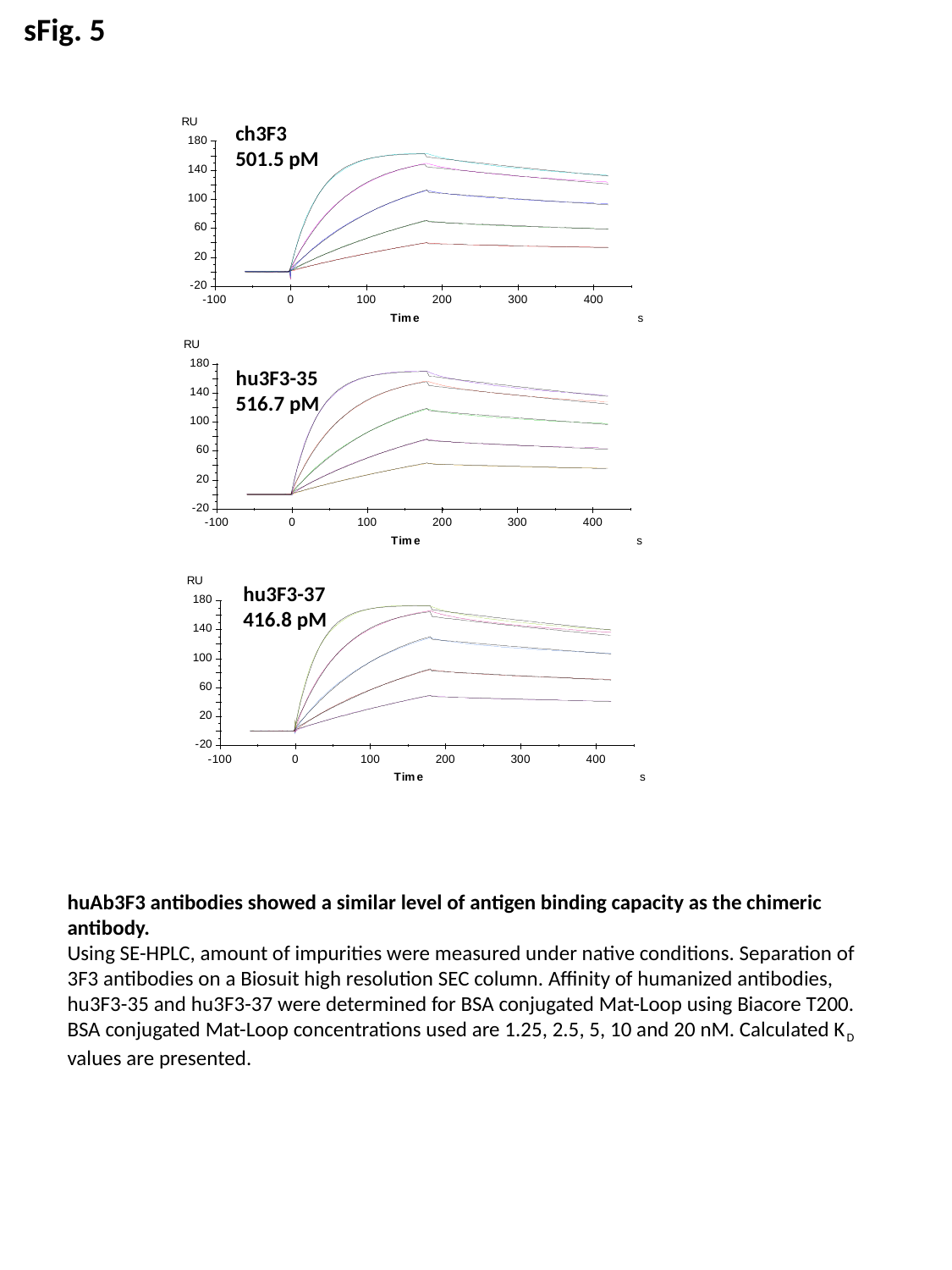

sFig. 5
ch3F3
501.5 pM
hu3F3-35
516.7 pM
hu3F3-37
416.8 pM
huAb3F3 antibodies showed a similar level of antigen binding capacity as the chimeric antibody.
Using SE-HPLC, amount of impurities were measured under native conditions. Separation of 3F3 antibodies on a Biosuit high resolution SEC column. Affinity of humanized antibodies, hu3F3-35 and hu3F3-37 were determined for BSA conjugated Mat-Loop using Biacore T200. BSA conjugated Mat-Loop concentrations used are 1.25, 2.5, 5, 10 and 20 nM. Calculated KD values are presented.
